# The Role of Fur in the Transcriptional and Iron Homeostatic Response of *Enterococcus faecalis*

**DOI:** 10.3389/fmicb.2018.01580

**Published:** 2018-07-17

**Authors:** Mauricio Latorre, Daniela Quenti, Dante Travisany, Kavindra V. Singh, Barbara E. Murray, Alejandro Maass, Verónica Cambiazo

**Affiliations:** ^1^Laboratorio de Bioinformática y Expresión Génica, Instituto de Nutrición y Tecnología de los Alimentos, Universidad de Chile, Santiago, Chile; ^2^Mathomics, Center for Mathematical Modeling, Universidad de Chile, Santiago, Chile; ^3^Center for Genome Regulation (Fondap 15090007), Universidad de Chile, Santiago, Chile; ^4^Instituto de Ciencias de la Ingeniería, Universidad de O’Higgins, Rancagua, Chile; ^5^Division of Infectious Diseases, Department of Internal Medicine, University of Texas Health Science Center at Houston, Houston, TX, United States; ^6^Center for Antimicrobial Resistance and Microbial Genomics, McGovern Medical School, University of Texas Health Science Center at Houston (UTHealth), Houston, TX, United States; ^7^Department of Mathematical Engineering, Universidad de Chile, Santiago, Chile

**Keywords:** ferric uptake regulator, *Enterococcus faecalis*, iron homeostasis, transcriptional networks, systems biology

## Abstract

The ferric uptake regulator (Fur) plays a major role in controlling the expression of iron homeostasis genes in bacterial organisms. In this work, we fully characterized the capacity of Fur to reconfigure the global transcriptional network and influence iron homeostasis in *Enterococcus faecalis*. The characterization of the Fur regulon from *E. faecalis* indicated that this protein (Fur) regulated the expression of genes involved in iron uptake systems, conferring to the system a high level of efficiency and specificity to respond under different iron exposure conditions. An RNAseq assay coupled with a systems biology approach allowed us to identify the first global transcriptional network activated by different iron treatments (excess and limited), with and without the presence of Fur. The results showed that changes in iron availability activated a complex network of transcriptional factors in *E. faecalis*, among them global regulators such as LysR, ArgR, GalRS, and local regulators, LexA and CopY, which were also stimulated by copper and zinc treatments. The deletion of Fur impacted the expression of genes encoding for ABC transporters, energy production and [Fe-S] proteins, which optimized detoxification and iron uptake under iron excess and limitation, respectively. Finally, considering the close relationship between iron homeostasis and pathogenesis, our data showed that the absence of Fur increased the internal concentration of iron in the bacterium and also affected its ability to produce biofilm. These results open new alternatives in the field of infection mechanisms of *E. faecalis*.

## Introduction

Iron is an essential metal required for several bacterial species (Chandrangsu et al., 2017). Taking advantage of its redox activity, different enzymes use this micronutrient as a cofactor in order to conduct numerous functions such as electron transfer, electrophilic catalysis, nitrogenase, and hydrogenase reactions (De Domenico et al., 2008). At elevated concentrations, iron can be toxic to the cell. Through Fenton and/or Haber–Weiss reactions, iron reacts with hydrogen peroxide and superoxide generating reactive oxygen species (ROS), which are able to damage cell membranes, proteins, and DNA (Eaton and Qian, 2002). In terms of pathogenesis, one of the principal response mechanisms of humans to confront bacterial infection is the seizure of free iron, which prevents the successful acquisition of this metal by pathogenic microbes. In an attempt to limit iron availability to pathogens during infection, the host increases the production of iron storage molecules in the gut, in order to reduce the bioavailability of this metal (Leon-Sicairos et al., 2015).

Changes in iron availability require complex control over mechanisms involved in iron homeostasis (De Domenico et al., 2008). In bacteria, the ferric uptake regulator (Fur) is the primary transcription factor controlling components related to iron homeostasis (Hantke, 2001; Chandrangsu et al., 2017). Fur was first described in *Escherichia coli* as an iron-responsive repressor of iron uptake systems. The mechanisms of action of this regulator are highly conserved in multiple bacterial pathogens (Carpenter et al., 2009; Lee et al., 2014; Ludwig et al., 2015; Pelliciari et al., 2015), including *Helicobacter pylori, Vibrio* sp., *Pseudomonas* sp., *Shigella flexneri*, and *Bacillus subtilis*. This transcriptional factor represses its target genes when the intracellular concentration of iron exceeds threshold levels (Fuangthong and Helmann, 2003). In the absence of the metal, Fur-mediated repression is lifted and the iron uptake genes are transcribed (Skaar, 2010) increasing the chances to capture the metal and recover homeostatic levels.

Besides its participation in iron metabolism, Fur contributes to pathogenesis, acting as a positive regulator of genes that encode virulence factors and proteins with roles in oxidative and pH-mediated stress responses, supporting the importance of Fur in the transcriptional regulation of several metabolic processes (Foster, 1991; Troxell and Hassan, 2013; Porcheron and Dozois, 2015). In this context, global gene expression analyses have revealed a novel role of Fur. This protein can function as a transcriptional activator or repressor through direct (promoter interaction) and indirect mechanisms (small regulatory RNAs) (Masse et al., 2007; Yu and Genco, 2012; Oglesby-Sherrouse and Murphy, 2013). In *E. coli*, besides transcriptional control over iron homeostasis systems, Fur regulates a total of 42 operons related to DNA synthesis, energy metabolism, and biofilm development, revealing the full transcriptional regulatory potential of this regulator (Seo et al., 2014). These data strongly support the fact that the absence of this regulator not only affects iron homeostasis, but also induces global cellular adjustments which impact several cell processes.

*Enterococcus faecalis* is a facultative anaerobe, Gram-positive bacterium, a common member of the gastro-intestinal tract microbiota. Since the early 90s, it has emerged as a major cause of local or systematic nosocomial infections, including urinary tract and abdominal infections, bacteremia, wound infections, and endocarditis (Arias and Murray, 2012; Guzman Prieto et al., 2016). In previous work, the first global transcriptional response of this bacterium to a non-toxic iron excess condition was described (Lopez et al., 2012). The data allowed authors to conclude that, in *E. faecalis*, iron acts as a complex stimulus capable of impacting diverse genes encoding for proteins involved in several metabolic processes, suggesting the presence of transcriptional regulators (among them Fur) which play a crucial role in coordinating gene expression under an iron excess condition.

Considering that *E. faecalis* is one of the principal hospital-acquired pathogens and the importance of the control of iron homeostasis during pathogenesis, we identified and characterized the transcriptional regulator Fur of *E. faecalis* in this article. To do this, Fur was removed from the bacterium, a strategy that allowed us to determine Fur gene targets. Fur regulon is limited to genes encoding for iron uptake proteins, unlike in other species, where Fur also controls the expression of genes involved in other metabolic processes. Next, using a systems biology approach, we determined the relevance of Fur over the global transcriptional response of *E. faecalis* under iron deficiency and excess conditions, showing that this bacterium has the capacity to reconfigure its gene network, activating others transcriptional regulators in order to maintain iron homeostasis.

## Results and Discussion

### Fur Regulon in *Enterococcus faecalis*

**FIGURE 1:**
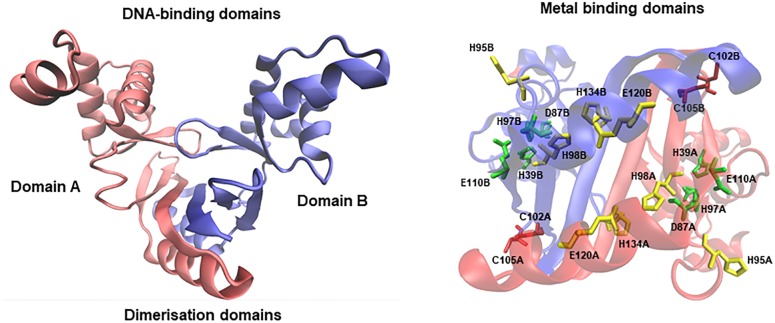
Homology model of Fur from *E. faecalis*. Red and blue chains structures denote domain A and B, respectively. Each domain includes from residues 10 to 142. The stereo view represents the three putative zinc/iron binding domains (conserve residues in red, green, and yellow).

Members of the Fur family of transcriptional regulators are conserved across bacterial species. In particular, the *E. faecalis* OG1RF genome contains three putative regulators in this family, Fur (iron homeostasis), Per (oxidative stress response), and Zur (zinc homeostasis) (Lopez et al., 2012). The Fur protein (149 aa) is encoded by a monocistronic gene (EF1525) highly conserved (more than 50% identity) across species of the *Lactobacillales* order (**Supplementary Figure S1**). Crystal protein models of Fur denote that this regulator acts as a dimer (Pecqueur et al., 2006; Sheikh and Taylor, 2009). Each chain possesses two main domains (A and B) involved in DNA-binding and dimerization; each monomer contains three putative metal binding sites, all of them able to coordinate zinc or iron atoms. In order to *in silico* validate the predicted gene annotation, we modeled Fur of *E. faecalis* using the closed conformation of Fur from *Vibrio cholerae* (over 30% identity and 50% similarity between both species’ protein sequences). The resulting model in *E. faecalis* describes an asymmetric dimer conformation with the presence of the DNA-binding and dimerization domains (**Figure 1**). In addition, each monomer contained three metal domains located in the same orientation as the crystal model of *V. cholerae*, conserving the specific residues involved in iron coordination. The coherent tertiary homology between the model and the crystal strongly suggests that the EF1525 gene from *E. faecalis* codes for the Fur transcriptional factor.

A previous *in silico* strategy identified three putative transport systems involved in iron homeostasis controlled by Fur in *E. faecalis*, the operons *fhuDCBG* (ferrichrome transporter), *feoAB* (ferrous iron transport), and *ycl*QPN (iron compound ABC transporter) (Latorre et al., 2014). To confirm if these three operons are direct targets of Fur, we generated a null deletion mutant of this protein (Δ*fur*) in *E. faecalis*, to later determine the transcript abundance of the three iron uptake operons between the wild type and the mutant strain. The qPCR assay showed that all iron uptake genes had at least a fivefold higher expression level in Δ*fur* than in the wild type strain of *E. faecalis* (**Table 1**). These results suggest that, in a growing media without adding (excess) or removing (deficiency) iron (i.e., control condition), Fur represses genes involved in iron uptake in *E. faecalis*, supporting the previous *in silico* prediction and its transcriptional regulatory action in this bacterium as described in other organisms (Troxell and Hassan, 2013).

**Table 1:** Changes in the relative abundance of Fur regulon genes.

Gene Id	Operon Id	Gene name	Gene annotation	qPCR (fold change Δ*fur*/WT)	Binding site
EF0188	EF0188	*fhuD*	Iron compound ABC transporter, substrate-binding protein	8 ± 1^∗^	TGAGAACGATTCTCA
EF0191	EF0191–EF0193	*fhuC*	Ferrichrome ABC transporter, ATP-binding protein	10 ± 1^∗^	TGATAACGATTCTCA
EF0192	EF0191–EF0193	*fhuB*	Ferrichrome ABC transporter, permease protein	9 ± 2^∗^	TGATAACGATTCTCA
EF0193	EF0191–EF0193	*fhuG*	Ferrichrome ABC transporter, permease protein	9 ± 1^∗^	TGATAACGATTCTCA
EF0475	EF0475–EF0476	*feoA*	Ferrous iron transport protein A	5 ± 1^∗^	TGAGAACGATTCTCA
EF0476	EF0475–EF0476	*feoB*	Ferrous iron transport protein B	6 ± 1^∗^	TGAGAACGATTCTCA
EF3082	EF3082–EF3085	*ycIQ*	Iron compound ABC transporter, substrate-binding protein	15 ± 3^∗^	TGAGAATGATTCTCA
EF3083	EF3082–EF3085	*ycIP*	Iron compound ABC transporter, ATP-binding protein	18 ± 3^∗^	TGAGAATGATTCTCA
EF3084	EF3082–EF3085	N/A	Iron compound ABC transporter, permease protein	16 ± 3^∗^	TGAGAATGATTCTCA
EF3085	EF3082–EF3085	*ycIN*	Iron compound ABC transporter, permease protein	12 ± 2^∗^	TGAGAATGATTCTCA


*Values were determined by qPCR and expressed as the fold change between the fur mutant (Δfur) and wild type strain. Asterisks represent significant differences (REST2008).*

### Iron Homeostasis Effects of Fur Absence

Iron homeostasis can be defined as a healthy control of the internal concentrations of this metal (De Domenico et al., 2008). In order to determine the influence of Fur over iron homeostasis in *E. faecalis*, we evaluated if the absence of the Fur impacted the viability of the bacterium growing under different scenarios of iron exposure.

**FIGURE 2:**
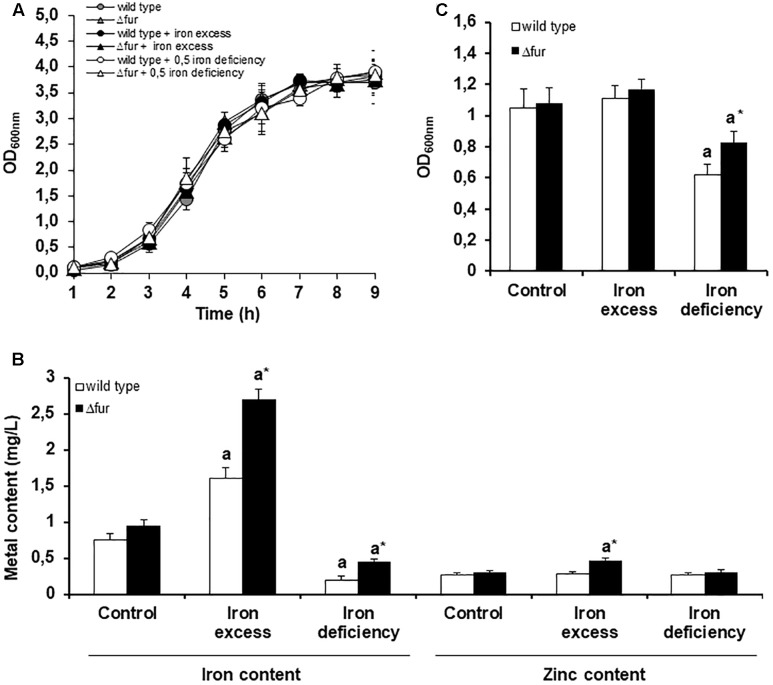
Homeostatic response of *E. faecalis* wild type and Δ*fur* (Fur mutant) under different iron conditions. **(A)** Growth curve of *E. faecalis* wild type and Δ*fur* under three iron exposure conditions. **(B)** Biofilm assay, optic density was measured after 24 h. **(C)** Bacterial metal content measured after 3 h of iron exposure. Letter a denotes significant difference between the control media and iron excess or deficit for the same strain. Asterisk (^∗^) marks significant difference between the wild type and Δ*fur* for the same growing condition of iron. Error bars represent standard deviation (SD) values. Three biological replicates (Mann–Whitney test, *p* < 0.05).

No differences in the growth rate were observed between the wild type and Δ*fur* strains (**Figure 2A**), suggesting that the absence of Fur does not impact the viability of the bacterium, even when exposed to an excess or a deficit of the metal (**Supplementary Figure S2**). This result is consistent with results in other bacterial species, where the removal of Fur has minor effects on growth *in vitro* (Pasqua et al., 2017). Same phenotype occurs with other metal regulators in *E. faecalis*, such as CopY (copper homeostasis), where the deletion of this transcription factor does not affect the viability of the bacterium under different copper treatments (Reyes-Jara et al., 2010).

As shown in **Figure 2B**, exposure of wild type and Δ*fur* mutant strain to excess iron resulted in a significant increase in the internal concentration of iron and zinc. On the contrary, iron deficit reduced the internal concentrations of iron and zinc. Considering that the mutant strain was constantly expressing the iron uptake systems, an increase in the internal concentration of iron was expected. In particular, the increase in zinc in the mutant strain exposed to iron excess can be explained as a requirement of this essential micronutrient as a cofactor in several oxidative stress enzymes and transcriptional factors. A similar phenotype occurs when the bacterium is exposed to other metals with redox activity (Latorre et al., 2014).

The capacity of *E. faecalis* to produce biofilm is directly correlated with the ability to colonize organs (Nallapareddy et al., 2006; Anderson et al., 2015). In order to study the participation of Fur in the pathogenesis of *E. faecalis*, a biofilm assay was performed growing the wild type and (*fur* strains at different conditions of extracellular iron. As shown in **Figure 2C**, under iron deficit, the (*fur* strain compared to wild type showed an increase in the production of biofilm. Previous information indicates that Fur mutants in *Pseudomonas aeruginosa* under low concentrations of iron are able to organize a more mature biofilm, a response likely triggered by an indirect signal effect produced by changes in the internal concentration of the metal (Banin et al., 2005). As showed in **Figure 2B**, (*fur* strain contained a higher internal concentration of iron compared to wild type following growth of both under the deficit condition. This difference may be triggering the higher production of biofilms as noted in *P. aeruginosa*.

### Transcriptional Response Activated in Absence of Fur

**FIGURE 3:**
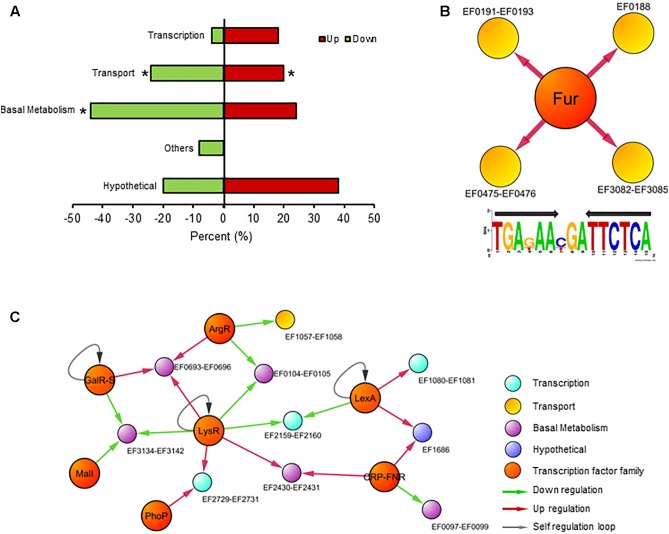
Transcriptional response in the absence of Fur. **(A)** Global transcriptional analysis of *E. faecalis* wild type and Δ*fur* growing in the iron control condition. RNAseq differentially expressed genes classify by COG super-class categories. Color bars represent the upregulated or downregulated genes in Δ*fur* compared to the wild type. Asterisk denotes significant enrichment in the bacterial genome (*p* (0.05) compared to total proportion. **(B)** Fur regulon of *E. faecalis*. Consensus logo of Fur regulon created with Fur DNA sequence found in promoters of EF0188, EF0191–EF0193, EF475–EF0476, and EF3082–EF3085 operons (the black arrow indicates palindromic sequence). **(C)** The graph contains 23 nodes (14 operons, 8 transcription factor families) connected by 26 edges (putative binding sites). Color circles represents COG super-class categories.

As mentioned, besides the transcriptional control over genes participating in iron homeostasis, Fur target genes also encode proteins involved in basal metabolism, energy production, and pathogenesis, classifying this regulator as a global transcription factor in several bacteria (Quatrini et al., 2007; Troxell and Hassan, 2013; Seo et al., 2014). To identify new targets of Fur in *E. faecalis*, we performed RNAseq comparing the global transcriptional abundance between the wild type and Δ*fur* strain, both growing in a control media without an iron excess or deficit condition. Besides the components involved in iron uptake, 65 transcripts significantly changed in abundance (40 upregulated and 25 downregulated) in the absence of Fur (putative genes targets of Fur, **Supplementary Table S1**). As expected, transport, and basal metabolism were over-represented in relation to the total functions present in the genome, highlighting genes coding for proteins involved in DNA replication, metal transport, and stress response (**Figure 3A**).

The next step was to examine whether the classic DNA-binding site sequence of Fur, a palindrome of 15 nucleotides (TGAnAAnnnTTnTCA), was present in the set of 65 genes differentially expressed in the Δ*fur* strain. As mentioned, this sequence is present in all the promoter regions from iron uptake operons in *E. faecalis*. In a recent article, six new DNA Fur binding motifs were identified in *E. coli* (Seo et al., 2014). These new DNA Fur binding motifs from *E. coli* (including the canonical Fur binding site) were searched on the promoter regions of the 65 putative genes targets of Fur. None of these new motifs were found on the first 300 bp upstream from the set of putative Fur target genes, suggesting that this set of 65 genes likely respond to secondary effects induced by the removal of Fur (e.g., an increase in internal iron concentration) and not to direct regulation by this transcription factor. This result suggests that Fur in *E. faecalis* possessed a high degree of specificity to regulate genes involved in iron homeostasis (*feo, fhu*, and *ycl* operons) (**Figure 3B**). This characteristic is in concordance with previous results obtained in CopY and Zur of *E. faecalis*, where these two metal regulators are exclusively confined to control the expression of genes involved in copper and zinc homeostasis, respectively (Latorre et al., 2015).

This result suggests the presence of other transcriptional factors controlling these 65 genes, probably activated in order to counteract a putative secondary effect induced by the absence of Fur. In order to identify putative regulators controlling the transcription for these components, the list of differentially expressed genes was integrated into the global regulatory network (**Figure 3C**). The resulting model contains 7 transcription factor families and 10 transcriptional units (10 operon, 29 genes), covering almost 50% of total transcriptional changes.

Members of the LysR (basal metabolism) and LexA (DNA repairs) families are classic regulators activated during stress conditions (Schell, 1993; Butala et al., 2009), probably sensing in the global regulatory model the increase in the internal iron concentration present in Δ*fur*. In addition, transcription factors related to general metabolism also are represented in the network. For example, the CRP–fumarate nitrate reductase (FNR) family modulates gene expression encoding for L-serine dehydratase (EF0097–EF0098). This enzyme participates in several process such as cell wall biosynthesis, amino acid synthesis and even host colonization, which principal characteristic is utilized by pyridoxal 5′-phosphate (PLP) or [Fe-S]cluster as cofactors (Thoden et al., 2014). In addition, some members of the CRP–FNR family of regulators also contain this iron cluster (Kiley and Beinert, 2003). The production and assembly of [Fe-S] clusters are indirectly controlled by Fur action (Roche et al., 2013), supporting the configuration of the transcriptional network model showed in **Figure 3C** to *E. faecalis*.

The absence of Fur impacted the transcriptional regulation of the bacterium in two ways. A direct regulation of the genes involved in iron uptake (three-independent systems), that comprise the Fur regulon in *E. faecalis*. The upregulation of these genes explained the increase in internal iron concentration produced in Δ*fur* compared to the wild type strain. This change did not disturb the viability of the bacterium, however, it did induce transcriptional changes of genes related to basal metabolism and DNA repair, indicating a secondary effect of the absence of Fur in *E. faecalis*. In particular, the operons EF1218–EF1223 and EF3204 encoding for spermidine/putrescine transports and cobalamin synthesis proteins, respectively, were upregulated in the Δ*fur* strain (**Supplementary Table S1**). Both systems participate in oxidative stress protection in bacterial species (Tkachenko et al., 2012; Ferrer et al., 2016), these genes are induced in *E. faecalis* probably as a response to the increase in internal iron concentration observed in the mutant compared to the wild type strain. In addition, the gene *nifU* (EF2391) was also is induced in the Δ*fur* strain. The protein NifU provides the molecular scaffold for the assembly of [Fe-S] clusters (Dos Santos et al., 2004). The induction of *nifU* in the mutant strain reaffirms a previous observation from the network model, that Fur in *E. faecalis*, besides its participation in the regulation of iron uptake genes, also has an indirect effect over the transcriptional activation of genes encoding for [Fe-S] proteins.

### Global Transcriptional Regulatory Network Activated in the Absence of Fur Under Different Iron Conditions

**FIGURE 4:**
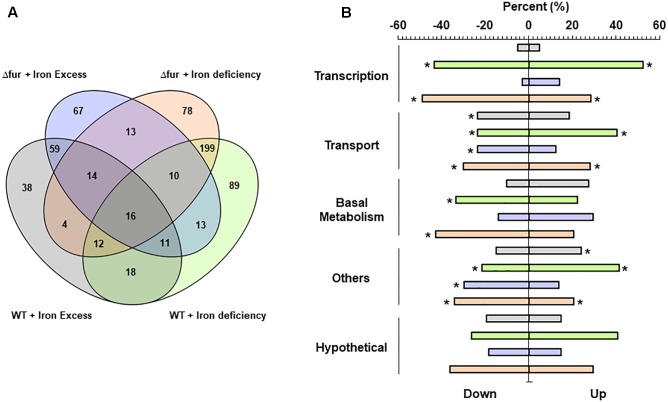
Global transcriptional analysis of *E. faecalis* wild type and Fur mutant growing at different iron treatments. **(A)** Venn diagrams. Numbers indicate the total genes activated on each condition. **(B)** RNAseq differentially expressed genes classify by COG super-class categories. Color bars represent the two *E. faecalis* strains growing under iron excess or deficiency. Asterisk denotes significant enrichment (*p* (0.05) compared to the total proportion in the bacterial genome.

Finally, the capacity of *E. faecalis* to reconfigure its global transcriptional response in order to cope with the absence of Fur under different scenarios of iron availability was tested. First, global expression data were obtained from *E. faecalis* wild type and Δ*fur* growing under two different iron treatments, deficiency (0.5 mM BDP for 3 h) and excess (0.5 mM FeCl_3_ for 3 h) (**Figure 4A**). In general, the global transcriptional response between the wild type and the mutant was similar after being exposed to the same iron treatments, highlighting the upregulation or downregulation of genes coding for proteins involved in transcription (i.e., gene regulators and nucleotide synthesis), basal metabolism and transport under the iron deficiency condition. A core of 16 genes changed in transcriptional abundance (with respect to the wild type strain growing in control media) under all iron treatments in both strains. In terms of the impact of the absence of Fur on the global expression of *E. faecalis* growing under iron excess condition, a total of 67 genes responded in this condition. On the other hand, during deficiency of the metal, 78 genes changed their transcript abundance (48 upregulated and 30 downregulated) in the Δ*fur* mutant strain only. In the deficit scenario, the absence of Fur primarily activated nucleotide pathways (**Figure 4B**), which is consistent with the high number of transcriptional factors activated in this condition.

The exposure to 0.5 mM of iron during 3 h induced changes in a total of 172 and 203 genes in the wild type and Δ*fur* strain, respectively. The global gene expression of *E. faecalis* OG1RF wild type treated with a concentration of 0.5 mM of iron during 6 h of exposure has been previously described (Lopez et al., 2012). This chronic non-toxic iron treatment activates a total of 475 genes, increasing internal iron content more than four times compared to the control growing condition. As showed in **Figure 2B**, the wild type strain treated with 0.5 mM of FeCl_3_ for 3 h duplicated the internal concentration of iron. The internal amount of iron in Δ*fur* tripled under the same condition. These significant differences in the internal concentration were correlated with an increase in the number of genes activated, where *E. faecalis* likely responds to a gradual rise in the secondary effects generated by excess iron, such as oxidative stress.

In previous work, the global response of *E. faecalis* to defibrinated horse blood was described using an experimental condition where the bioavailability of iron was reduced, homologating a metal deficiency scenario. Interestingly, from the total activated genes during the exposure to 0.5 mM BDP in the wild type and Δ*fur*, 40% (158 genes) and 50% (176 genes), respectively, changed their transcript abundance during the blood exposure. These results suggest that, under an iron deficiency scenario, *E. faecalis* activates an important number of common genes, which may be involved in similar global metabolic adjustments in order to maintain the viability and iron homeostasis of the bacterium.

In relation to other bacteria, global expression analysis in *Caulobacter crescentus* and the human pathogen *Vibrio vulnificus* growing in iron-limited conditions, indicate that most of the observed changes belong to genes participating in transport, highlighting the TonB and TomB system (Alice et al., 2008; da Silva Neto et al., 2013). In particular, these protein complexes are located in the outer membrane, which is absent in Gram + bacteria such as *E. faecalis*. Nevertheless, *E. faecalis* activates a large set of ABC transporters under the iron deficiency condition, a complementary mechanism to Fur regulon that is also able to incorporate iron into the cell (Moeck and Coulton, 1998; Koster, 2001). *E. coli* and *Geobacter sulfurreducens* have a similar global transcriptional response to different iron concentrations showed by *E. faecalis*, activating an important number of genes involved in transport and basal metabolism, components mainly participating in energy production (McHugh et al., 2003; Embree et al., 2014). Another global gene expression study performed in the pathogen *Pasteurella multocida*, indicates that iron deprivation prompts the expression of a component involved in antibiotic resistance (penicillin-binding proteins) (Paustian et al., 2001). During the iron deficit scenario, *E. faecalis* wild type and Δ*fur* upregulated the genes EF1055, EF1943, and EF2068, all encoded for drug resistance proteins (**Supplementary Table S1**). In addition, virulence genes in *E. faecalis*, such as collagen adhesion protein (*ace-*EF1099), glucose starvation protein (*gls24*-EF0079), and the cell wall surface anchor family (*Ebp* system-EF1092–1093) are up or downregulated under the different iron conditions both in the wild type and Δ*fur* strains.

**FIGURE 5:**
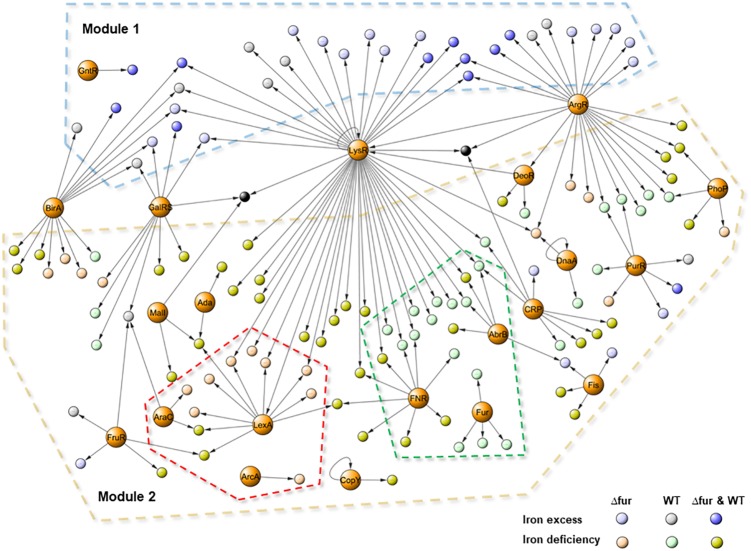
Transcriptional regulatory network activated by iron excess and deficiency in *E. faecalis* wild type and Δ*fur* (Fur mutant). The graph contains 153 nodes (139 activated operons and 21 family of regulators) connected by 181 edges (putative binding sites). Color circles represent differentially expressed responses of each operon in the RNAseq experiment. Dash lines indicate: Module 1 (iron excess) in light blue, Module 2 (iron deficiency) in light brown, wild type sub-network in light green and Δ*fur* in light pink.

**Figure 5** presents a global transcriptional regulatory network activated by different conditions of iron in the wild type and Δ*fur*. The model contains a total of 181 edges connecting 153 nodes, 21 regulator families, and 139 operon (330 total genes included), covering 60% of the total differentially expressed genes obtained from the RNAseq assays. The topological analyses describe a consistent and reliable network, with an in-degree coefficient value of 3.19 showing a classical power law distribution present in other biological networks (Albert, 2005) and also present in previous models activated by copper and/or zinc in *E. faecalis* (Latorre et al., 2014, 2015).

Based on the number of gene targets, the network contained three types of transcriptional factors. Global regulators (LysR, ArgR, GalRS, and BirA), controlling 10 or more operons coding for several metabolic process, primarily related to basal metabolism. Local regulators, controlling the expression of a small number of genes coding for proteins participate in the same processes (Ada, DeoR, DnaA, ArcA, GntR, CopY, and Fur), and the rest of the regulator families can be classified as master regulators, controlling between 5 and 10 gene targets functionally related to each other. In terms of transcriptional activation, the model had two main modules. Module 1 contained genes activated during the iron excess condition and Module 2 contained genes activated during the deficit of this metal. Global regulators control genes from both modules; in particular, the ArgR (arginine metabolism) and LysR (general metabolism) families were also present in copper and zinc network models (Latorre et al., 2014, 2015). These regulator families have been shown to respond to different stimuli, explaining the activation under different metal conditions, which is also extremely important to control the global structure of the network.

The model contained two operons activated under all the studied conditions (black nodes in the center of the model). Both operons, EF3134–EF3142 (PTS system) and EF0764 (hypothetical protein), had high regulation complexity, and were controlled by at least three global regulators which may explain induction under iron excess and deficit. In addition, PTS systems have also been correlated with the virulence capacity of *E. faecalis* (Sauvageot et al., 2017). Activation in different iron conditions opens an interesting field to study the relationship between pathogenesis, metal homeostasis, and gene regulation in *E. faecalis*.

It has been demonstrated that the transcriptional network activated during copper excess is characterized by the activation of two modules, one specifically related to copper detoxification (*cop* operon) and a second one involved in general metabolism (Latorre et al., 2014). During iron excess, the activation of Module 1 was mainly controlled by global regulators, supporting the requirements of energy production and synthesis of basal components. As opposed to copper, the specific mechanism involved in iron detoxification in bacteria is currently unclear. Non-specific bivalent cation transporters such as cation diffusion facilitators (CDF) may play a role, however, no transcriptional regulatory factors controlling this element were identified in the network presented in **Figure 5**.

With respect to the differences between the numbers of genes activated by iron excess or deficit in both strains, Module 2 present this information covering the mayor proportion of the network. In addition to the global regulators, this module contained a set of families of transcriptional factors which were in turn controlling genes induced by iron excess, such as CRP, PurR, FruR, and Fis. These families are classified as global regulators participating in basal metabolism, energy production and DNA topology, and were also activated during the zinc and copper treatments, supporting participation in metal metabolism. Inside Module 2, it was possible to identify two sub-networks. The first one was composed of genes activated in the wild type during iron deficiency (dashed green line in the model). Besides the activation of the four operons involved in iron uptake controlled by Fur, this module contained the regulator FNR, a major repressor of anaerobic metabolism that contains an oxygen-responsive [Fe-S] cluster (Carpenter and Payne, 2014). The inactivation of this protein lies in [Fe-S] stability; the intracellular iron deprivation generated by BDP likely impacted the amount of this metal to produce this cluster, explaining the activation of the gene target of FNR present in the sub-network.

The second sub-network of genes activated only in Δ*fur* by iron deficiency described the specific reconfiguration of the network in order to cope with the absence of Fur. Inside this sub-network, the presence of LexA, which controls the greatest number of genes principally involving in DNA repair and oxidative stress damage, was highlighted. Disruption of LexA in *Cyanobacterium synechocystis* increases the expression of genes involved in iron uptake (Kizawa et al., 2016). The same phenotype occurred in *E. faecalis* Δ*fur*, supporting the participation of LexA as a compensative transcriptional mechanism during the iron deficiency condition.

## Conclusion

The transcription factor Fur is one of the most studied regulators in bacterial species. Recently, it has been established that Fur is not restricted to iron uptake systems, but is also involved in the control of genes coding for DNA synthesis, energy metabolism, and biofilm formation. Thus, the importance of this regulator beyond the iron homeostasis is clear. In this work, we presented a full transcriptional and metal homeostatic characterization of Fur in *E. faecalis*. The *in silico* approach validated through experimental expression analyses indicated that the unique genes controlled by Fur are part of the iron uptake mechanism.

Unlike other bacterial species, *E. faecalis* allocates specific metal regulators (such as Fur, CopY, and Zur) to the transcriptional control of proteins related to metal homeostasis, conferring to the system a high level of efficiency and specificity under different metal availability scenarios. Interestingly, the results showed that the absence of Fur affects internal iron concentration and activated iron uptake systems. The absence of Fur also impacted the internal concentration of other micronutrients (like zinc) and induced the transcriptional activation of the *cop* operon (involved in copper homeostasis). These findings strongly suggest cross-talk between iron, copper and zinc at different homeostatic levels.

The systems biology approach allowed the identification of the first transcriptional regulatory network activated by iron in bacterial species described to date. The iron excess condition primarily activated genes and regulators related to basal metabolism. This response was increased when Fur was deleted, activating more elements principally involved in energy production and [Fe-S] proteins, a response that is consistent with the necessity to control the increase of intracellular iron in the mutant strain. On the other hand, iron limitation induced a large set of ABC transporter, optimizing iron acquisition through alternative uptake mechanism such as iron-siderophore transporter.

Besides the direct influence over iron uptake mechanisms, the removal of Fur from the system activated other regulators (which also responds to changes in copper availability), denoting a complex transcriptional reconfiguration in this bacterium to cope with the absence of Fur under different iron availability scenarios, and supporting the idea of a transcriptional interplay between different metals. A core of 16 genes changed in transcriptional abundance under all iron treatments conditions in both strains. This set of elements can be classified as general iron response genes, which are able to respond to changes in the intracellular concentration of the metal, most of which code for transporters (ABC and PTS) and hypothetical proteins.

In *P. aeruginosa* and *Staphylococcus aureus*, iron deficiency leads to the activation of several virulence factors (Kim et al., 2003; Oogai et al., 2011). On contrary, virulence factor gene induction is directly correlated with an increase in iron concentration in *Pseudomonas syringe* (Kim et al., 2009). In *E. faecalis*, the complex response of the virulence genes under the different iron conditions is consistent with current knowledge, supporting that there is no a direct relationship between the transcriptional response of bacterial virulence factors and iron availability.

The high number of genes changing their transcript abundance, in particular during the iron deficiency condition, coupled with the activation of new gene regulators, supports the idea that the effects of the loss of control in iron uptake by the removal of Fur lead the bacterium to reconfigure its global transcriptional regulation. In this context, the final analysis involved the integration of the RNAseq data into the global transcriptional regulatory network of *E. faecalis*, a strategy which allowed us to identify common and specific gene regulatory mechanisms that were activated in all studied cases.

Finally, bearing in mind that *E. faecalis* is one of the principal hospital-associated pathogens, the characterization of Fur in this bacterium opens a new alternative to study the participation of this regulator in pathogenesis. Likewise, considering the direct relationship between iron homeostasis and bacterial infection, the transcriptional network models presented in this work provide a list of putative candidates (genes and regulators) involved in iron adaptation, providing new alternatives to study other mechanisms related to pathogenesis in *E. faecalis*.

## Materials and Methods

### *Enterococcus faecalis* Fur Deletion

*Enterococcus faecalis* non-polar deletion mutant of *fur* was constructed using the PheS^∗^ system (Kristich et al., 2007). Two fragments of ca. 1000 bp located upstream and downstream of target gene were amplified by PCR using: up region: fur_up_s CGCGCGGCCGCTTGTGGATGTTGGCTTACCA and fur_up_a ACCATGAAAATACAGCTCCTTGTTTAGAATGATTACA; down region: fur_down_s AGGAGCTGTATTTTCATGGTATTTGTCAAAGTTGT and fur_down_a CGCGTCGACACTAGCCCAACAATGTTGGC primers. The amplicon was first cloned in pGEM-T Easy (Promega) and then inserted in pCJK47. The resulting constructs were transferred to *E. faecalis* OG1RF (wild type strain) by electroporation (single cross over insertions). The loss of the plasmid was selected on medium MM9YEG agar supplemented with 10 mM p-Cl-Phe and X-gal 200 lg/ml. Mutant strains (*E. faecalis* OG1RF Δ*fur*) were verified by sequencing (deletion area) and pulsed with field gel electrophoresis.

### Culture Conditions, Growth Curves, and Biofilm Assay

*Enterococcus faecalis* OG1RF (wild type) and Δ*fur* were grown in N medium (Peptone 1%, yeast extract 0.5%, Na_2_HPO_4_ 1%, and glucose 1%) (Reyes-Jara et al., 2010). For all experiments, strains were independently cultured overnight in N medium broth at 37°C. The next day, each strain was diluted in parallel cultures to a final OD_600 nm_ of 0.05 and then grown at 37°C and 160 rpm. For growth curves, iron cultures were supplemented with concentrations of 0 (iron normal), 0.25, 0.5, 1, 2, 5, 10, and 15 mM of FeCl_3_ (iron excess), and 0.25, 0.5, 1, 2, 5, 10, and 15 mM of 2,2′-bipyridyl iron chelator (BPD, iron deficiency) in order to determine non-lethal conditions. Bacterial growth was monitored each hour over 8 h and at 24 h by OD_600 nm_. Biofilm assay were carried out as previously described (Mohamed et al., 2004). Briefly, strains were grown overnight, diluted 1:100 in 200 μl N medium and inoculated into polystyrene microtiter plates. After 24 h of static incubation at 37°C, attached cells from plates were processed, fixed with Bouin’s fixative for 30 min, stained with 1% crystal violet for 30 min, and rinsed with distilled water. OD_600 nm_ was then determined. Each assay was performed in triplicate.

### Intracellular Metal Content

After the iron treatments (control, deficiency or excess), the intracellular metal content for each culture was determinate as described below (Latorre et al., 2011). Both strains growing in the different iron conditions and at the same growth stage (mid-exponential, 3 h of growth cultures according to the growth curves), were centrifuged and then suspended in 200 μl of HNO_3_ (Merck) and digested during 24 h at 65°C. After the acid lysis, total metal composition was determinate by total reflection X-ray fluorescence (TXRF). The data were expressed as microgram per liter, normalized per total cell culture (absorbance) and represent the average value of three measurements for independent biological replicates. The statistical analysis was done using an ANOVA test (*p* < 0.05).

### Transcriptomic Experiments

For RNAseq assays, total RNA was isolated from three replicate cultures from each strain grown to mid-exponential phase (OD_600 nm_ ∼ 1.5) in N medium (control) and supplemented with 0.5 mM of FeCl_3_ or 0.5 mM of 2,2′-bipyridyl using the RNeasy kit (Qiagen) as previously described (Reyes-Jara et al., 2010). For RNAseq analysis, each Illumina FASTQ library was first processed by FASTX to remove low complexity and low quality reads. EDGE-pro v1.3.1 software (Magoc et al., 2013) was used to align the reads to *E. faecalis* OG1RF complete genome^1^ using Bowtie2 (Langmead and Salzberg, 2012). The gene expression estimation was directly obtained from the alignment output. Protein translation table with coordinates of protein coding genes (.ptt) files were generated using in-house perl scripts and were used as inputs into EDGE-pro. In order to generate the RPKM file, EDGE-pro was run with default parameters except for the read length (-l101). Samples were concatenated through edgeToDeseq.perl script provided by EDGE-pro. DESeq was used to identify the gene expression level. Differentially expressed genes between the wild type and Δ*fur* strains growing in the control condition, and wild type or Δ*fur* 0.5 mM of FeCl_3_ and 0.5 mM of 2,2′-bipyridyl were considered significant based on a *p*-value ≤ 0.01 and FDR of 2. Complete data is accessible in the **Supplementary Information**.

All qPCR assays and data analyses were conducted as described in previous work (Reyes-Jara et al., 2010). All experiments were carried out in triplicate using three-independent RNA samples. The results were expressed as the fold change between wild type strain and Δ*fur*. qPCR expression values were normalized using the *gdh* reference gene (EF1004). Statistical analyses were assessed using REST2009 (Pfaffl et al., 2002).

### Bioinformatics

Fur protein homologous in *Lactobacillales* order organisms were obtained using BlastP from the National Center for Biotechnology Information (NCBI) website (template *E. faecalis* Fur EF1525). Global Fur alignments were performed using Clustal W (Hirosawa et al., 1995). The Fur molecular 3D model was generated by SWISS-MODEL program (Guex and Peitsch, 1997), using the crystallographic model information from *V. cholerae* Fur (PDB Id 2W57) as a template. The final model was displayed with VMD v1.9.4 software (Humphrey et al., 1996).

Transcriptional regulatory networks activated by the different status of iron in *E. faecalis* wild type and Δ*fur* were performed as previously described in the construction of copper and zinc activated networks. Briefly, each global expression result from RNAseq experiments were integrated into a global transcriptional regulatory network model from *E. faecalis* and experimentally validated (EfaecalisGTN.gbk file) (Latorre et al., 2014). To relate information between the expression data and the global network we used graphic displays performed using Cytoscape software (Shannon et al., 2003) (**Supplementary Table S2**). All network topology analyses were carried out using R software (iGraph package).

## Author Contributions

ML designed the research and analyzed the data. DQ and DT generated the microbiological and RNAseq data, respectively. AM and KS supported the bioinformatics and mutagenesis analyses, respectively. ML, VC, and BM wrote the paper. ML has responsibility for the manuscript. All authors read and approved the final content.

## Conflict of Interest Statement

The authors declare that the research was conducted in the absence of any commercial or financial relationships that could be construed as a potential conflict of interest.

## Footnotes

1 https://www.ncbi.nlm.nih.gov/nuccore/NC_017316.1

## Supplementary Material

FIGURE S1 Protein sequence alignments between *E. faecalis* Fur (EF1525) and Fur archetypes from different bacterial species. Color circles denote conserve motif involved in iron/zinc binding.

FIGURE S2 Growth curve of *E. faecalis* wild type and Δ*fur* under different conditions of iron exposure (excess and deficiency) in three biological replicates (Mann–Whitney test, *p* < 0.05). Error bars represent standard deviation (SD) values.

TABLE S1 Differentially expressed genes in *E. faecalis* wild type and Δ*fur*. The table shows the differentially expressed genes obtained from the RNAseq experiments.

TABLE S2 Global transcriptional regulatory network activated by iron deficiency or excess in *E. faecalis* wild type and Δ*fur*.

DATA SHEET S1 RNAseq complete data.
