# Supplementary figures and images for: The Role of Fur in the Transcriptional and Iron Homeostatic Response of Enterococcus faecalis
Source: Front Microbiol. 2018 Jul 17;9:1580. doi: 10.3389/fmicb.2018.01580 (PMC6056675; doi:10.3389/fmicb.2018.01580)

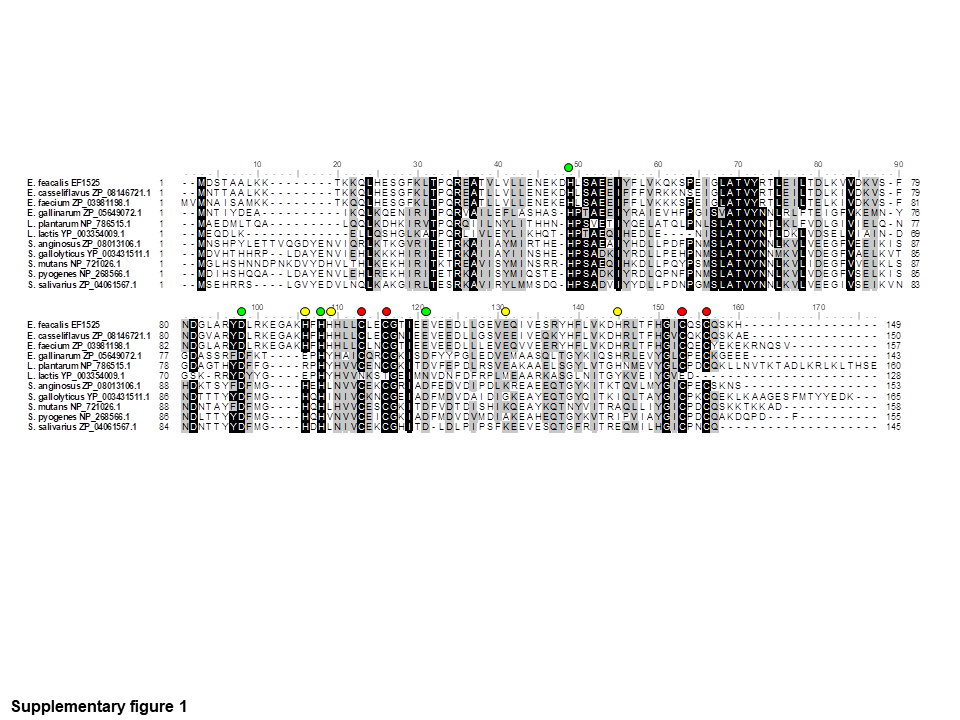

Supplement: FIGURE S1 — Protein sequence alignments between E. faecalis Fur (EF1525) and Fur archetypes from different bacterial species. Color circles denote conserve motif involved in iron/zinc binding. [file Image_1.TIF]

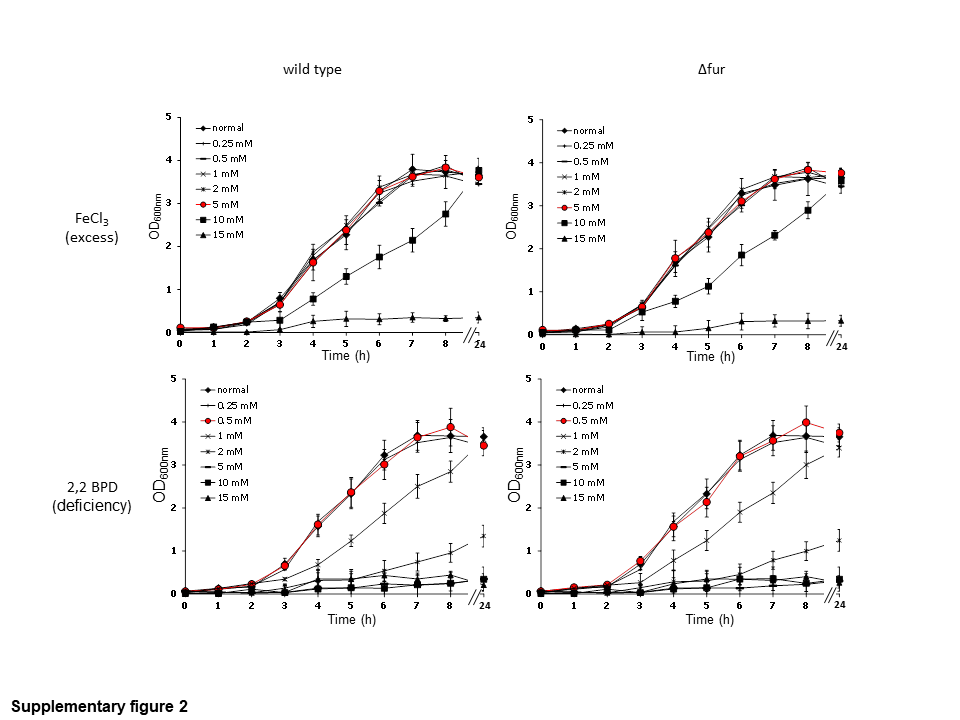

Supplement: FIGURE S2 — Growth curve of E. faecalis wild type and Δfur under different conditions of iron exposure (excess and deficiency) in three biological replicates (Mann–Whitney test, p < 0.05). Error bars represent standard deviation (SD) values. [file Image_2.TIF]
